# Markers of fertility in reproductive microbiomes of male and female endangered black-footed ferrets (*Mustela nigripes*)

**DOI:** 10.1038/s42003-024-05908-0

**Published:** 2024-02-23

**Authors:** Sally L. Bornbusch, Alexandra Bamford, Piper Thacher, Adrienne Crosier, Paul Marinari, Robyn Bortner, Della Garelle, Travis Livieri, Rachel Santymire, Pierre Comizzoli, Michael Maslanka, Jesús E. Maldonado, Klaus-Peter Koepfli, Carly R. Muletz-Wolz, Alexandra L. DeCandia

**Affiliations:** 1 Center for Conservation Genomics, Smithsonian’s National Zoo & Conservation Biology Institute, Washington, DC USA; 2 Department of Nutrition Science, Smithsonian’s National Zoo & Conservation Biology Institute, Washington, DC USA; 3 Department of Biology, Georgetown University, Washington, DC USA; 4 Smithsonian-Mason School of Conservation, George Mason University, Front Royal, VA USA; 5 Center for Animal Care Services, Smithsonian’s National Zoo & Conservation Biology Institute, Front Royal, VA USA; 6 National Black-Footed Ferret Conservation Center, US Fish and Wildlife Service, Carr, CO USA; 7 Prairie Wildlife Research, Stevens Point, WI USA; 8 Biology Department, Georgia State University, Atlanta, GA USA; 9 Center for Species Survival, Smithsonian’s National Zoo & Conservation Biology Institute, Front Royal, VA USA

**Keywords:** Microbial ecology, Conservation biology

## Abstract

Reproductive microbiomes contribute to reproductive health and success in humans. Yet data on reproductive microbiomes, and links to fertility, are absent for most animal species. Characterizing these links is pertinent to endangered species, such as black-footed ferrets (*Mustela nigripes*), whose populations show reproductive dysfunction and rely on ex-situ conservation husbandry. To understand microbial contributions to animal reproductive success, we used 16S rRNA amplicon sequencing to characterize male (prepuce) and female (vaginal) microbiomes of 59 black-footed ferrets at two ex-situ facilities and in the wild. We analyzed variation in microbiome structure according to markers of fertility such as numbers of viable and non-viable offspring (females) and sperm concentration (males). Ferret vaginal microbiomes showed lower inter-individual variation compared to prepuce microbiomes. In both sexes, wild ferrets harbored potential soil bacteria, perhaps reflecting their fossorial behavior and exposure to natural soil microbiomes. Vaginal microbiomes of ex-situ females that produced non-viable litters had greater phylogenetic diversity and distinct composition compared to other females. In males, sperm concentration correlated with varying abundances of bacterial taxa (e.g., *Lactobacillus*), mirroring results in humans and highlighting intriguing dynamics. Characterizing reproductive microbiomes across host species is foundational for understanding microbial biomarkers of reproductive success and for augmenting conservation husbandry.

## Abstract (web summary)

Genital microbiomes of black-footed ferrets are associated with reproductive outcomes, such as non-viable offspring and sperm quality, and differ between wild and ex-situ environments.

## Introduction

Reproductive microbiomes are traditionally defined as the communities of microbes inhabiting the reproductive tract^1,2^. In humans and other animals, distinct microbial communities have been identified in both sexes at multiple sites along the reproductive tract (e.g., vaginal, labial, preputial, urethral, and seminal), with variation in the bacterial diversity, composition and taxonomic membership^1–4^. Reproductive microbiomes are increasingly recognized for their roles in nearly every stage of reproduction. There is growing evidence that reproductive microbiomes can influence conception rates, mediate maternal health during pregnancy, and shape infant development (as reviewed in refs. ^3,4^). Although this evidence stems mainly from the study of humans and agricultural animals, investigating reproductive microbiomes may be particularly valuable for endangered species that rely on conservation breeding for species survival. Here, we present correlations of reproductive microbiomes and markers of fertility in both males and females of an endangered species, the black-footed ferret (*Mustela nigripes*; hereafter, black-footed ferret or ferret). Our dual goals for this study are to (a) expand our understanding of factors that shape reproductive microbiomes in wildlife species and (b) provide valuable data on correlations with reproductive outcomes that can be used to inform conservation breeding strategies.

Most commonly, vaginal microbiomes have been studied for mediating vaginal health (e.g., pathogen resistance, epithelial maintenance) and colonizing neonates^5^. In humans, most healthy vaginal microbiomes are dominated by *Lactobacillus, L. crispatus* in particular, a trait that is, so far, unique to humans^6^. The abundance of *Lactobacillus* is thought to facilitate a micro-environment (e.g., low pH) that promotes probiotic bacteria and simultaneously repels pathogens^6^. The vaginal microbiomes of non-human animals are typically more diverse, with *Lactobacillus* members being rare or absent. Nevertheless, these animal vaginal communities often contain abundant lactic acid producing bacteria that may provide similarly beneficial functions as *Lactobacillus*^4^. For example, in captive Coquerel’s sifakas (*Propithecus coquereli*), vaginal microbiomes harbored virtually no *Lactobacillus* but did include numerous, abundant members of the Lactobacillales order of lactic acid bacteria^7^. Lactic acid production is only one of many mechanisms by which vaginal microbes can promote vaginal health, with other functions including the competitive exclusion and direct inhibition of pathogens and the maintenance and repair of vaginal epithelium^8–11^. Whereas *Lactobacillus* members appear to perform these functions in humans, the apparent rarity of this genus in the vaginal microbiomes of most non-human animals suggests that other microbes may also be able to fulfill the same beneficial roles. These patterns suggest that there are multiple avenues by which vaginal microbiomes can be taxonomically structured to promote vaginal health across different animal hosts.

In multiple species, the composition of vaginal microbiomes has been linked to reproductive status and outcomes^12^. In humans and non-human primates, vaginal microbiome structure is shaped by reproductive state, varying significantly between ovarian cycle phases^13^. In humans, bacterial vaginosis is characterized by increased vaginal diversity and decreased *Lactobacillus* abundance, which are strongly linked to an increased risk of pre-term birth^14,15^. In domestic dogs (*Canis lupus familiaris*), the presence of specific genera, such as *Staphylococcus*, *Pasteurella*, or *Corynebacterium*, in vaginal microbiomes is correlated with significantly increased risk of still-born puppies^16^. While vaginal microbiome structure may vary across species, there is far less variation within species and disruption or imbalance in these communities beyond the normal range of intraspecific variation may negatively affect reproductive outcomes.

Compared to research on female reproductive microbiomes, studies in males are sparse. Outside of humans and rodent models, semen microbiomes have been investigated mainly in agricultural animals^17–19^ with a dearth of studies in wildlife species. In humans, semen microbiomes have been correlated with sperm quality and motility (as reviewed in ref. ^20^). Namely, multiple studies have reported that increased abundance of *Lactobacillus* in semen was linked to improved sperm characteristics whereas enrichment for *Prevotella* correlated with negative sperm markers^21–23^. Collecting semen, however, is often an invasive process, making it difficult to study in endangered or wild species. When semen is unavailable, microbial samples are often taken from the foreskin or prepuce to characterize male reproductive microbiomes. In free-ranging rhesus macaques (*Macaca mulatta*), for example, prepuce microbiomes showed high inter-individual variation, varied across age groups, and included certain, abundant bacterial genera also found in human semen microbiomes^24^. In collared peccaries (*Pecari tajacu*), prepuce microbiomes included abundant *Corynebacterium* and *Staphylococcus*, with increased abundances of *Corynebacterium* correlating with decreased sperm membrane activity^25^. Notably, very little is known about how male reproductive microbiomes vary across animal populations and environments and their subsequent impact on reproductive health.

In the present study, we characterize the reproductive microbiomes of black-footed ferrets, an endangered carnivore endemic to North America. Having gone through a severe population bottleneck in the late 1900s, the species was thought to have gone extinct by 1979 but was rediscovered in 1981^26,27^. Since then, significant conservation efforts, including captive-breeding programs at multiple facilities, coupled with annual reintroductions, have successfully bolstered ex-situ and wild populations, with an estimated ~300–500 individuals in natural habitats^28^. However, there are continuing reproductive concerns in ex-situ populations, including low conception rates, stillbirths (non-viable offspring), neonatal deaths, maternal and neonate infections, and poor sperm quality in males^29–31^. Wolf et al. ^31^ reported that, in healthy male ferrets of reproductive age, 55–58% failed to sire offspring across two years. Over the past 10 years at the Smithsonian’s National Zoo and Conservation Biology Institute (NZCBI), an average of 35% of females did not whelp successfully (ranging from 31.5% to 50%). Across females that did whelp, an average of 85% of kits survived (ranging from 68% to 98%). Together, these data suggest relatively low fertility but high infant survival in ex-situ ferrets. In addition, continuous reintroductions of new individuals have been needed to maintain nearly every wild population. In 2022, it was estimated that there are only ~150 breeding adults living in the wild, a mere 10% of the estimated 1500 breeding adults needed to consider down-listing the species’ endangered status^28^. This indicates that reproductive success may be limited for reintroduced and/or wild individuals, likely from a combination of disease susceptibility^28^, low genetic diversity and inbreeding depression^32^, and low whelping rates and offspring survival^29^. Whether microbiomes contribute to these patterns of reproduction remains unknown, but it has been hypothesized that inbreeding depression may influence animal microbiomes through combined interactions with the host’s reduced genomic capacity and potentially diminished immune competency^33^. Addressing this gap is a crucial component of understanding black-footed ferret reproduction and health, with implications for conservation breeding strategies. For example, characterizing the reproductive microbiomes of ex-situ and wild ferrets in the context of reproductive success can provide the foundations for targeted approaches to microbial therapies such as pre- and probiotics or transfaunations. It can further provide opportunities to apply multi-omic approaches to conservation breeding strategies that previously relied solely on host genomic and health data.

To characterize variation in black-footed ferret reproductive microbiomes, and their potential role in reproductive outcomes, we performed 16S rRNA amplicon sequencing on male (prepuce) and female (vulvovaginal, hereafter referred to as vaginal) microbiomes of ferrets living at two ex-situ facilities and in the wild (Supplementary Fig. 1). Ferrets were housed at NZCBI (male = 17, female = 12) or the National Black-footed Ferret Conservation Center in Carr, Colorado (FCC; male = 23, female = 13), or living in the wild at Conata Basin in Buffalo Gap National Grassland in Wall, South Dakota (Conata; male = 6, female = 8). Notably, unlike the majority of wild black-footed ferret populations, the Conata Basin population is considered self-sustaining, not having had any reintroductions in ~23 years. This population thus provides an important baseline for the microbiomes of a successfully reproducing wild population of black-footed ferrets.

We assessed the bacterial taxonomic membership and explored patterns of diversity (alpha diversity) and composition (beta diversity). The strengths of our dataset lie in our ability to test for sex-based variation in ferrets across multiple environments and analyze correlations between microbiome structure and markers of fertility such as number of viable and non-viable offspring in females and sperm concentration in males. Given that host-associated microbiomes are influenced by both internal (e.g., physiology) and external (e.g., environment, diet) factors, we hypothesized that black-footed ferret reproductive microbiomes would vary between sexes and environments and reflect variation in markers of fertility. Based on previous studies of non-human animals^24^, we expected that reproductive microbiomes would vary between the sexes, with female vaginal microbiomes showing less inter-individual variation compared to male prepuce microbiomes. Although there are few previous studies examining variation in reproductive microbiomes between environments, studies of other bodily microbiomes (e.g., skin and gut microbiomes) demonstrate variation between ex-situ vs in-situ populations^34–36^. We thus predicted that ferret reproductive microbiomes would differ between the ex-situ and wild ferrets, with more minor differences between the two ex-situ populations. We further expected to find signals of correlations between markers of fertility and components of ferret microbiomes. Namely, we expected that female ferrets that produced non-viable offspring would show uncharacteristically high vaginal diversity and distinct composition, mirroring patterns seen across other species described above. We further expected specific bacterial taxa to correlate with male sperm concentration. However, given the species-specific nature of most animal-associated microbiomes, we did not expect the same microbes found to correlate with sperm characteristics in humans or model animals to be the same microbes correlating in ferrets.

## Results

### Reproductive microbiomes vary by sex and location across all ferrets

**Fig. 1:**
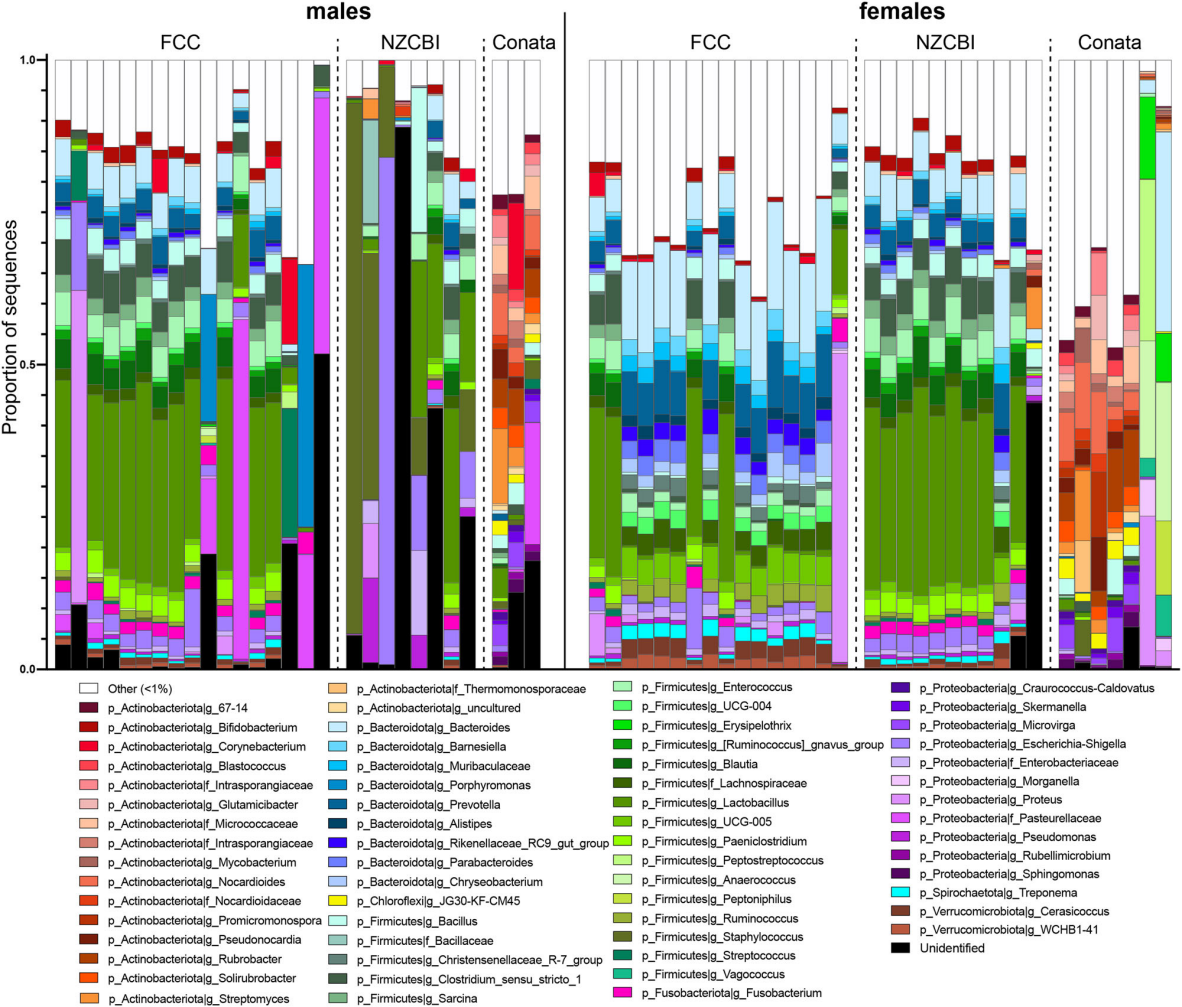
Genus-level membership (relative abundances) in black-footed ferret (*Mustela nigripes*) reproductive microbiomes. Ferret males and females from two ex-situ facilities (FCC and NZCBI) and the wild (Conata) (NZCBI; male = 8, female = 11, FCC; male = 17, female = 13, Conata Basin; male = 3, female = 7). Genera are identified by color and labeled with the phylum and deepest taxonomic assignment. Taxa representing <1% of the microbiomes were combined into the category “Other”. “Unidentified” represents taxa that were identified as bacteria but not assigned to a phylum.

After sequencing and bioinformatic filtering, the full dataset included 59 individuals (NZCBI; male = 8, female = 11, FCC; male = 17, female = 13, Conata Basin; male = 3, female = 7, Supplementary Table 1). We generated a total of 549,676 sequences (mean = 9317, range =  2943–83,723) representing 2478 amplicon sequence variants (ASVs) assigned to 473 genera in 24 phyla. At the phylum level, ferret reproductive microbiomes were dominated by Firmicutes (mean = 41.9%, range = 0–88.1%) and Bacteriodota (16.4%, 0–52.8%), with additional substantial contributions from Proteobacteria (14.0%), and Actinobacteriota (12.3%) (Fig. 1). Notably, wild ferrets of both sexes in Conata Basin harbored higher abundances of Actinobacteriota compared to ferrets at both ex-situ sites (wild, mean = 48.3%; FCC, mean = 3.7%; NZCBI, mean = 6.9%), while ex-situ ferrets harbored higher abundances of Firmicutes (wild, mean = 17.0%; FCC, mean = 46.7%; NZCBI, mean = 47.5%). Across all samples, there were 20 abundant genera (>1% of sequence reads), with *Lactobacillus* (mean = 12.8%) having the greatest relative abundance. At the ASV level, 13 ASVs were abundant across all samples (>1% of sequence reads in both sexes), with two *Lactobacillus* ASVs showing the greatest relative abundances (ASV6068; mean = 5.4%, ASV6106; mean = 4.8%).

When testing for variation in membership, we identified 175 ASVs and 105 genera that were statistically differentially abundant between the two sexes using analysis of compositions of microbiomes with bias correction (ANCOMBC; Supplementary Fig. 2)^37^. Of the ASVs, 174 were statistically structural zeros, indicating that the taxon was absent or nearly absent from at least one sex. The other ASV, a member of the *Bacteroides* genus, had a W statistic of 159. Similarly, all but one of the genera were structural zeros, with the one having a W statistic of 102 (a member of the *WD2101-soil-group* genus). Of the ASVs, 19 were unidentified at phylum level, 18 were members of the genus *Bacteroides*, and 12 were members of the genus *Christensenellaceae R-7 group*. The *Bacteroides* and *Christensenellaceae R-7 group* ASVs were more abundant in female microbiomes compared to males (*Bacteroides*: female, mean = 7.4%, maximum = 32.6%; male, mean = 3.4%, maximum = 7.9%; *Christensenellaceae R-7 group*: female, mean = 0.7%, maximum = 2.8%; male, mean = 0.0%, maximum <0.1%).

**Fig. 2:**
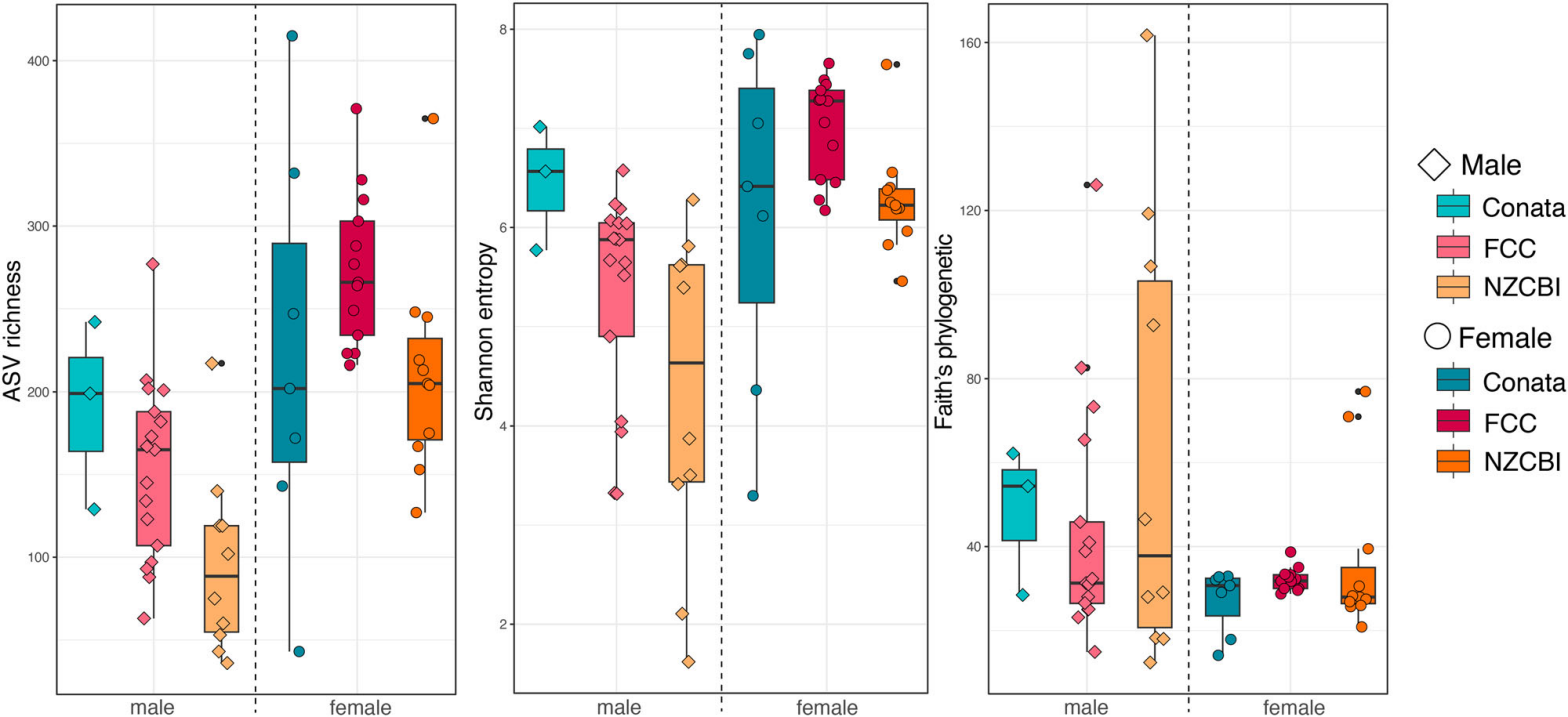
Variation in alpha diversity in black-footed ferret (*Mustela nigripes*) reproductive microbiomes. Alpha diversity (observed features, Shannon diversity, and Faith’s phylogenetic diversity) of reproductive microbiomes in male and female black-footed ferrets reproductive microbiomes from two ex-situ facilities (FCC and NZCBI) and the wild (Conata) (NZCBI; male = 8, female = 11, FCC; male = 17, female = 13, Conata Basin; male = 3, female = 7).

When using ANOVAs of linear models (LMs) with sex, location, and their interaction as fixed effects, the diversity (i.e., alpha diversity) of reproductive microbiomes varied by sex and location (Table 1; Fig. 2). The interaction between sex and location was significant for Shannon diversity but not for ASV richness or Faith’s phylogenetic diversity (Table 1). Sex alone was significantly associated with ASV richness and Faith’s phylogenetic diversity, with females generally having greater ASV richness and males have greater Faith’s phylogenetic diversity (Fig. 2). Location alone was only significantly associated with ASV richness (Table 1; Fig. 2).

**Table 1:** Results of ANOVAs (Sum of squares Type II and/or Type III for unbalanced sample design) for linear models of alpha diversity testing for variation in black-footed ferret (*Mustela nigripes*) reproductive microbiomes

		ASV richness		Shannon diversity				Faith’s phylogenetic diversity	
		Sum of squares Type II		Sum of Squares Type III		Sum of squares Type II		Sum of squares Type II	
		*F*	*p* value	*F*	*p* value	*F*	*p* value	*F*	*p* value
Full	sex (df: 1,53)	41.46	<0.0001	0.18	0.666			7.28	0.009
	location (df: 2,53)	6.07	0.004	2.10	0.131			1.82	0.171
	sex*location (df: 2,53)	1.89	0.161	4.72	0.012			0.91	0.406
Males	location (df: 2,28)	12.11	0.002			7.80	0.010	2.42	0.133
Females	location (df: 2,25)	2.33	0.115			2.75	0.081	1.29	0.290

The full model run on all samples included sex (male, female), location (ex-situ: FCC and NZCBI, in-situ: Conata), and their interaction as fixed effects, whereas the sex-specific models included only location as a fixed effect. Type III results were only reported when there was a significant interaction term in the model.

**Fig. 3:**
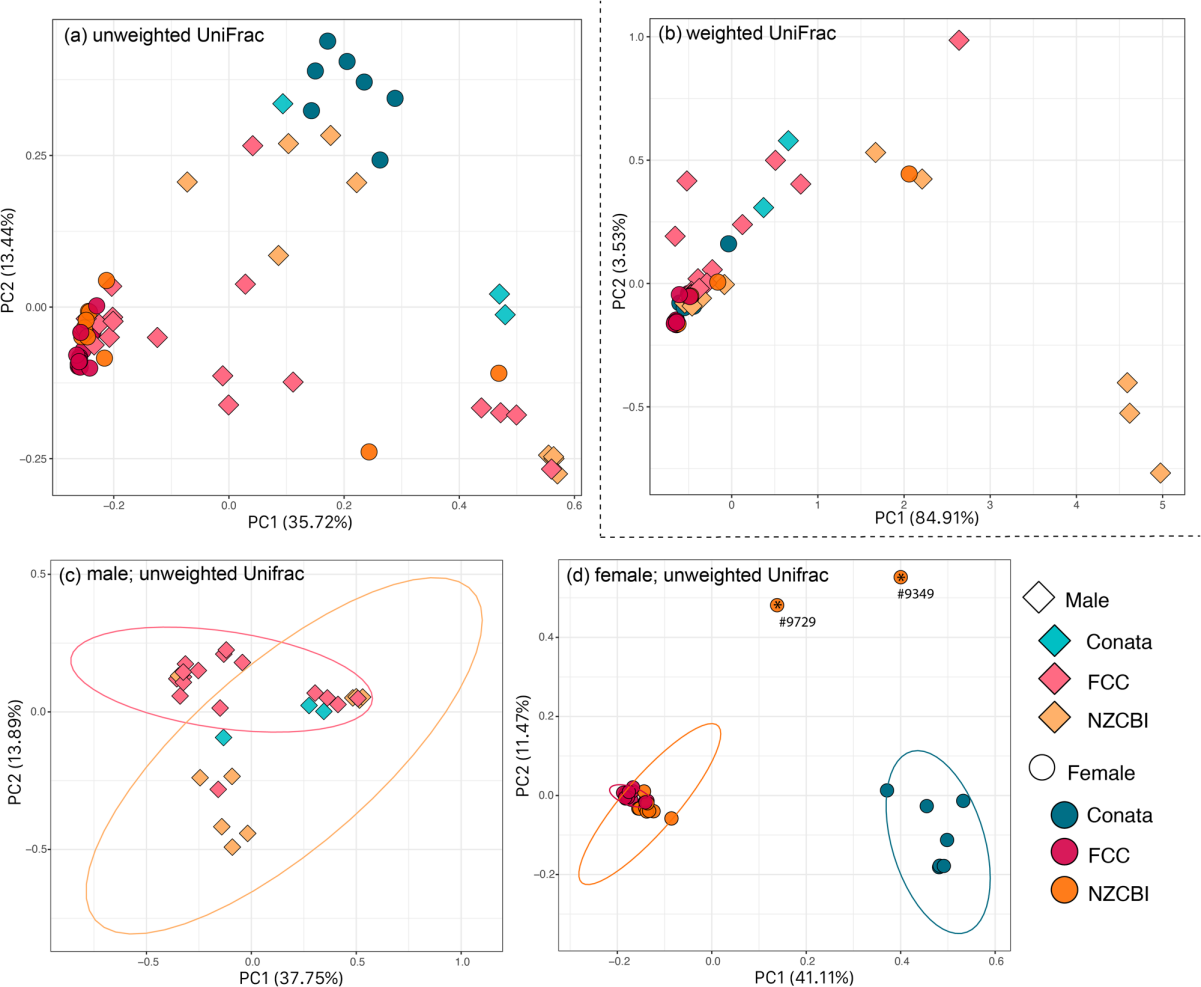
Principle coordinate analyses of bacterial beta diversity for reproductive microbiomes in male and female black-footed ferrets (*Mustela nigripes*). Ferrets were housed at two ex-situ facilities (FCC and NZCBI) and the wild (Conata) (NZCBI; male = 8, female = 11, FCC; male = 17, female = 13, Conata Basin; male = 3, female = 7). Unweighted UniFrac (**a**) and weighted UniFrac (**b**) distances for all samples, and unweighted UniFrac for sex specific variation in males (**c**) and females (**d**) with 95% confidence interval ellipses. Points noted with asterisks in (**d**) correspond to samples from the two females that produced entire litters of non-viable offspring.

When using permutational multivariate analysis of variance (PERMANOVA; R-Studio, adonis in {vegan} package) for bacterial community composition (i.e., beta diversity), we found that the interaction between sex and location was significant for both unweighted UniFrac (UUF) and weighted UniFrac (WUF) (Table 2), suggesting that microbial composition varied in a sex-specific manner across the three populations (Fig. 3).

**Table 2:** Results of PERMANOVAs models for beta diversity (unweighted and weighted UniFrac distances) testing for variation in black-footed ferret (*Mustela nigripes*) reproductive microbiomes

		Unweighted UniFrac PERMANOVA			Weighted UniFrac PERMANOVA		
		*R*^2^	*F*	*p* value	*R*^2^	*F*	*p* value
Full	sex*location (df: 2,53)	0.05	2.35	0.01	0.08	3.30	0.028
Males	location (df: 2,28)	0.18	2.92	0.003	0.22	3.65	0.037
Females	location (df: 2,25)	0.40	9.56	0.0001	0.12	1.91	0.051

The full model run on all samples included sex (male, female), location (ex-situ: FCC and NZCBI, in-situ: Conata), and their interaction as fixed effects, whereas the sex-specific models included only location as a fixed effect. PERMANOVA models assessed marginal effects such that for the full model, only the interaction is analyzed.

### Environment influenced reproductive microbiomes within each sex

To analyze the influence of environment on the microbiomes of each respective sex, we parsed the data into sex-specific subsets. The membership of ferret microbiomes varied across locations in both sexes (Fig. 1). In male ferrets, 143 ASVs were differentially abundant between the Conata and the two ex-situ populations and all were found to be structural zeros, indicating that the taxon was absent or nearly absent from at least one location (Supplementary Fig. 2). Between the two ex-situ populations, 119 ASVs were differentially abundant, all of which were structural zeros (Supplementary Fig. 2). The taxa with greatest representation across all of these ASVs included taxon that were unidentified at the phylum level (19 ASVs), and in the genera *Bacteroides* (9 ASVs), *Lactobacillus* (6 ASVs), *Prevotella* (6 ASVs), and *Staphylococcus* (6 ASVs). In female ferrets, 286 ASVs were found to be differentially abundant between the Conata and the two ex-situ populations, all of which were structural zeros (Supplementary Fig. 2). Only 88 ASVs were found to be differentially abundant between FCC and NZCBI, which were also all structural zeros (Supplementary Fig. 2). The genera with the highest representations among these ASVs included *Bacteroides* (25 ASVs), *Christensenellaceae R-7 group* (9 ASVs), *Lactobacillus* (9 ASVs), and *Prevotella* (9 ASVs). In both sexes, the *Lactobacillus* genera was more abundant in ex-situ ferrets compared to wild ferrets (FCC; mean = 12.8%, maximum = 33.3%, NZCBI; mean = 17.7%, maximum = 35.4%, Conata; mean = 1.0%, maximum = 5.0%). In contrast, the phylum Actinobacteriota was differentially abundant across locations in both sexes, with wild ferrets harboring greater abundances. There were 102 differentially abundant ASVs across locations in both sexes, suggesting that variation across environments was correlated with some similar taxa in both sexes.

We found that bacterial diversity varied across locations in male ferrets, but not female ferrets. In prepuce microbiomes, location was significantly correlated with ASV richness and Shannon diversity, with wild ferrets having greater diversity compared to ex-situ ferrets (Table 1). In contrast, across vaginal microbiomes, location was not significantly associated with any measure of alpha diversity (Table 1). UUF bacterial composition varied by environment in both male and female ferrets (Table 2). When visually comparing clustering of UUF distances, male prepuce microbiomes showed less distinct clustering by environment compared to female vaginal microbiomes, which clustered tightly by ex-situ and wild locations (Fig. 3). WUF, however, only varied significantly by location in male ferrets (Table 2), suggesting that females across environments had more similar abundance-weighted composition compared to males.

### Markers of fertility correlate with reproductive microbiomes in male and female ferrets

**Fig. 4:**
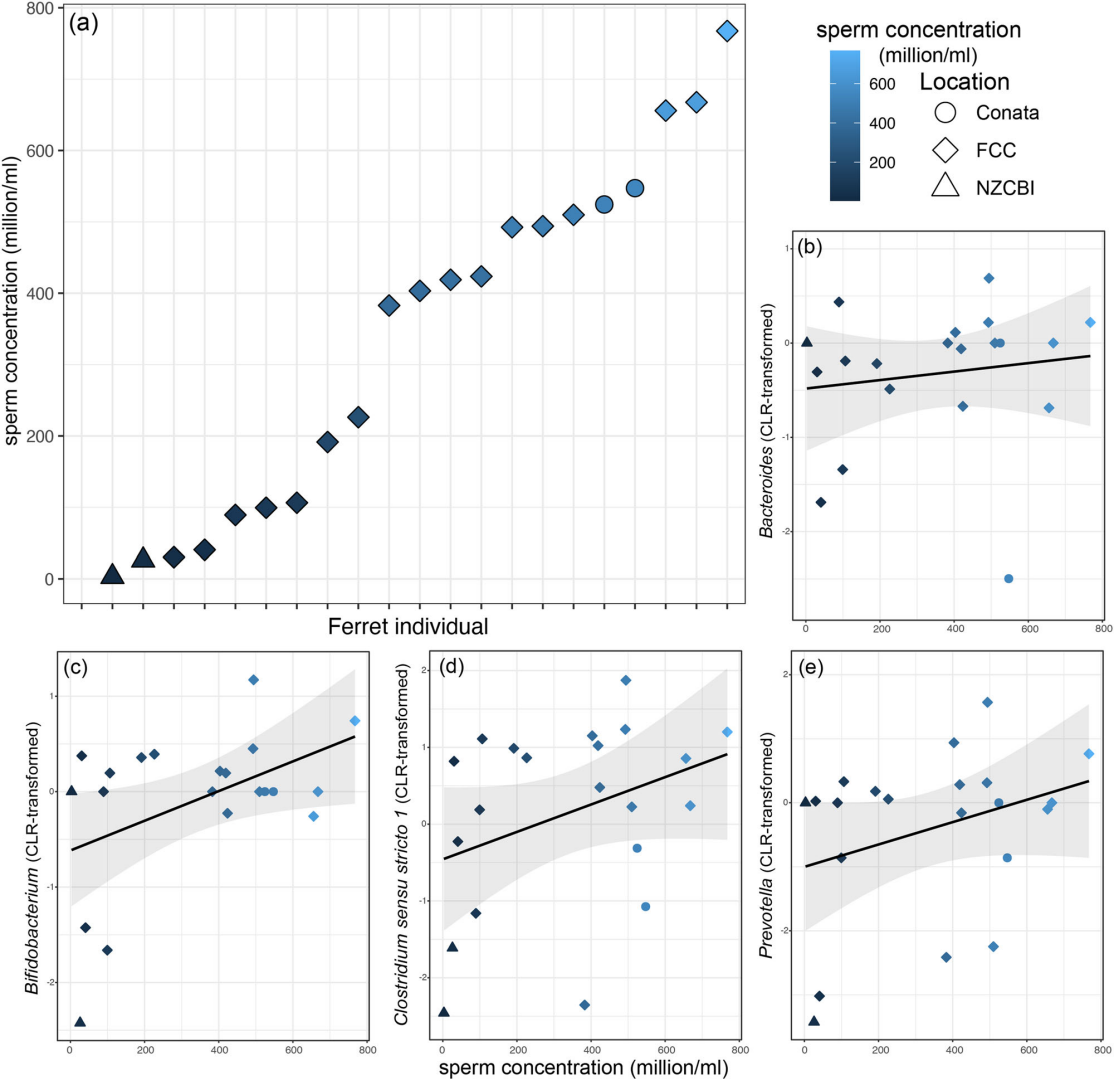
Variation in sperm concentration and bacterial taxa in the prepuce microbiomes of male black-footed ferrets (*Mustela nigripes*). Ferrets were housed at two ex-situ facilities (FCC and NZCBI) and the wild (Conata) (*n* = 21). Sperm concentration varies across individual ferrets (**a**) and correlations with differentially abundant bacterial genera (as calculated by ANCOMBC) visualized with linear trendlines (**b**–**e**).

When assessing reproductive traits in male ferrets across all three locations, sperm concentration (million cells per milliliter) varied from 3.0 mill/ml to 767.5 mill/ml (*n* = 21 males; Fig. 4). When examining whether sperm concentration was predictive of aspects of preputial reproductive microbiomes, we found no significant correlations with bacterial diversity or composition. ANCOMBC identified 128 ASVs that were statistically differentially abundant across sperm concentrations. These included 21 ASVs that were unidentified at the phylum level, and members of *Bacteroides* (9 ASVs), *Lactobacillus* (8 ASVs), *Bifidobacterium* (6 ASVs) and *Clostridium sensu stricto 1* (5 ASVs) (Fig. 4), with each of those genera showing increases in abundance with increasing sperm concentrations.

When assessing reproductive outcomes in female ferrets at FCC and NZCBI, the total number of live offspring produced prior to sampling ranged from 0 to15 (Supplementary Table 1). Across both facilities, 10 females produced viable offspring while 14 did not produce any offspring. Three NZCBI females produced non-viable offspring within 6 months of microbiome sampling: Ferret #9349 was sampled 115 days prior to producing a litter of 2 non-viable kits, ferret #9492 was sampled 83 days prior to producing a litter of 4 non-viable kits and 1 viable kit, and ferret #9729 was sampled 63 days prior to producing a litter of 4 non-viable kits. For both ferret #9349 and ferret #9729, the entire litter was non-viable.

**Fig. 5:**
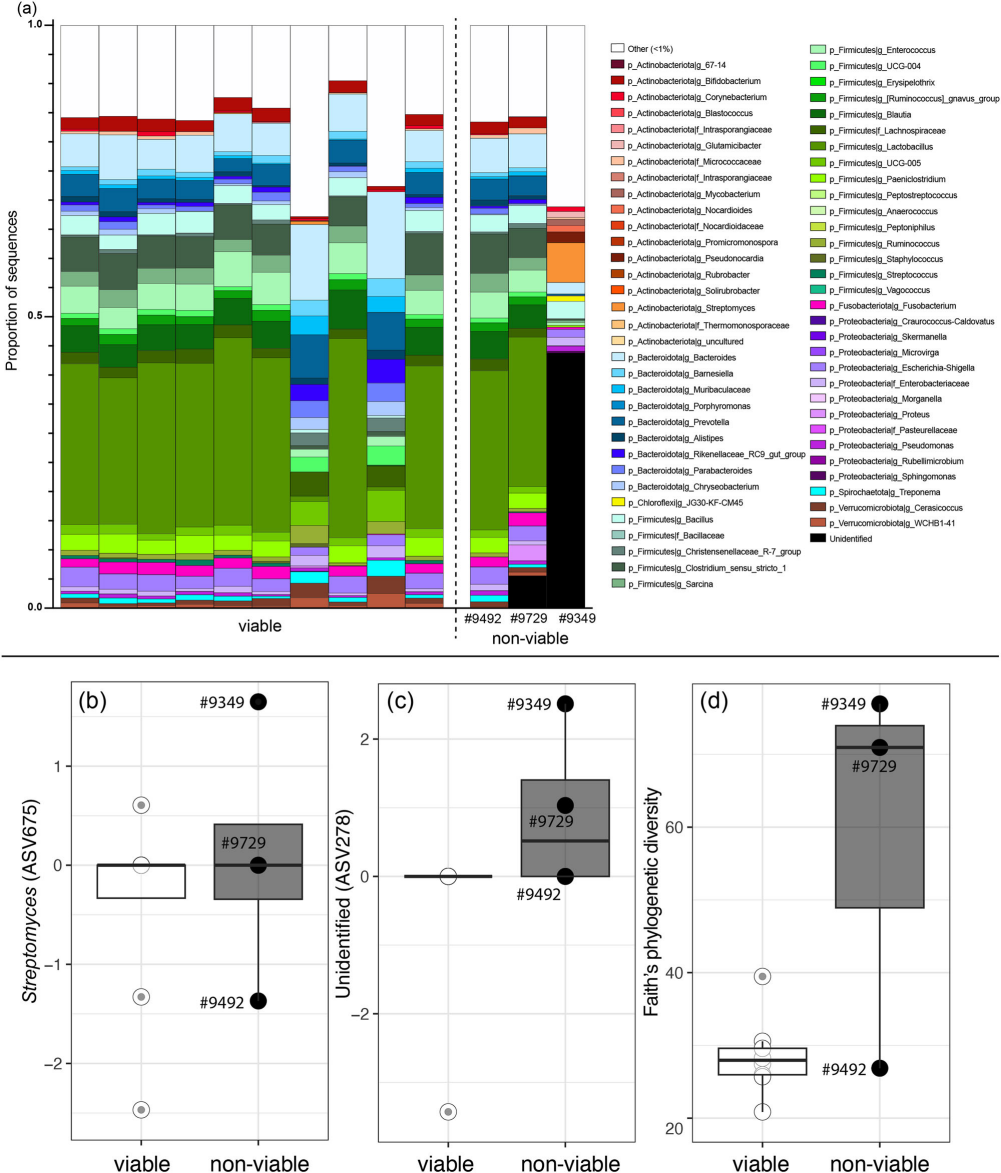
Membership and bacterial diversity of female vaginal microbiomes in black-footed ferrets (*Mustela nigripes*) that produced viable offspring (*n* = 10) or non-viable offspring within 6 months before or after sampling (*n* = 3). Genus-level membership (**a**) across female ferrets that produced viable offspring and for each of the three ferrets that produced non-viable kits. Genera are identified by color and labeled with the phylum and deepest taxonomic assignment. Taxa representing <1% of the microbiomes were combined into the category “Other”. “Unidentified” represents taxa that were identified as bacteria but not assigned to a phylum. Variation in differentially abundant amplicon sequence variants (ASVs) (**b**, **c**) and Faith’s phylogenetic diversity (**d**) between female ferrets that produced viable offspring and that produced non-viable kits. Points are labeled with the IDs of the two ferrets that produced entire litters of non-viable kits.

When examining whether diversity of vaginal microbiomes varied with these measures of reproductive outcomes in the subset of females with available data, we found that the number of live offspring was negatively correlated with Faith’s phylogenetic diversity (ANOVA Type II SS: *F*_(1, 21)_ = 6.55, *p* = 0.018), but was not correlated with ASV richness (ANOVA Type II SS: *F*_(1, 21)_ = 0.05, *p* = 0.822) or Shannon diversity (ANOVA Type II SS: *F*_(1, 21)_ = 0.02, *p* = 0.876). We further found that the number of non-viable offspring was positively correlated with Faith’s phylogenetic diversity (Fig. 5d; ANOVA Type II SS: *F*_(1, 21)_ = 12.75, *p* = 0.002), but not of ASV richness (ANOVA Type II SS: *F*_(1, 21)_ = 1.62, *p* = 0.216) or Shannon diversity (ANOVA Type II SS: : *F*_(1, 21)_ = 0.03, *p* = 0.207). This suggests that the viability of ferret offspring was predicted by the bacterial phylogenetic relationships of vaginal microbes, but not overall bacterial richness and abundance.

When examining vaginal microbiome composition, the number of non-viable offspring was significantly associated with both UUF and WUF distances (PERMANOVA: UUF, *F*_(1, 23)_ = 1.90, *R*^2^ = 0.071, *p* = 0.044; WUF, *F*_(1, 23)_ = 8.65, *R*^2^ = 0.242, *p* = 0.096). Number of live offspring was not significantly correlated with UUF or WUF (PERMANOVA: UUF, *F*_(1, 23)_ = 1.52, *R*^2^ = 0.059, *p* = 0.101; : WUF, *F*_(1, 23)_ = 0.159, *R*^2^ = 0.004, *p* = 0.887). The two ferrets whose entire litters were non-viable clustered distinctly from the rest of the individuals (Fig. 3; noted with asterisks). In contrast, the ferret who produced a litter of mixed non-viable and live offspring clustered with the rest of the female ferrets at her facility that produced live offspring.

The membership of the vaginal microbiomes between ferrets that did or did not produce non-viable offspring differed, with 218 ASVs that were differentially abundant, all of which were structural zeros. Namely, an ASV that was unidentified at the phylum level (ASV278) was significantly enriched in ferrets that produced non-viable offspring (Fig. 5a, c). Although this ASV was assigned to the Bacteria Kingdom (i.e., it included the conserved region of the 16S gene), it was not assigned to phylum in our taxonomy, nor could it be identified via NCBI BLAST. Another ASV in the genus *Streptomyces* (ASV675) was significantly increased in the ferrets that produced non-viable offspring (Fig. 5).

## Discussion

Using a comparative approach, we show that black-footed ferret reproductive microbiomes reflect the host’s sex, environment, and markers of fertility, even when considering a small number of individuals. Bacterial membership, diversity, and composition varied between prepuce and vaginal microbiomes, with wild ferrets differing from ex-situ ferrets in both sexes. Female ferrets that produced litters of non-viable offspring had distinct microbiomes compared to females that produced viable offspring, suggesting an interplay between reproductive microbiomes and reproductive success. In male ferrets, sperm concentration was correlated with varying abundances of certain taxa, supporting similar links between fertility and human male reproductive microbiomes. Although our limited samples sizes for wild individuals limits the scope of our interpretations, these patterns reinforce the need to expand the study of reproductive microbiomes across animal species and incorporate such research into animal management and conservation breeding programs^33,38,39^, examples of which we discuss below.

Between the two sexes, female vaginal microbiomes had greater bacterial richness, but lower phylogenetic diversity compared to males, suggesting richer but more phylogenetically similar communities across females. Within these females, the diversity of ferret vaginal microbiomes did not differ across environment, which contrasted with the result that diversity varied across environments within the male ferrets. Similarly, WUF beta diversity of vaginal microbiomes did not vary by location within females, but did vary significantly within males. Together, these results suggest that ferret vaginal microbiomes may be more phylogenetically constrained within and across populations compared to male prepuce microbiomes. This mirrors previous findings in macaques that female microbiomes are less variable compared to male reproductive microbiomes^24^. In female humans, shifts in vaginal microbiomes have been definitively linked to infection susceptibility and reproductive complications such as preterm or stillbirths^5^, whereas variation in male human reproductive microbiomes is, thus far, only loosely linked to fertility^20^. This pattern suggests that, compared to male ferret microbiomes, female vaginal microbiomes may be more tightly constrained to specific microbial taxa that preserve reproductively relevant functions of the community (e.g., pathogen resistance). While small sample sizes, particularly for males, limit our interpretations to the individuals and populations studied here, these results, within the context of existing literature, provide an interesting hypothesis for further testing the variability and stability of male and female reproductive microbiomes across black-footed ferrets and other at-risk wildlife species.

With our comparative approach across different locations, we find evidence that reproductive microbiomes may reflect the ferret’s environmental conditions in both males and females. Similar to findings in other host-associated microbiomes (e.g., gut and skin), reproductive microbiomes were distinct between ex-situ and in-situ populations. Environmental variation in host-associated microbiomes has been linked to differences in diet (particularly in gut microbiomes)^40–42^, social interactions^43–45^, disease^46,47^, and, notably, exposures to environmental microbiomes^48,49^. In previous studies of ex-situ endangered primates that either free-ranged in natural habitat enclosures or were housed in indoor enclosure, animals that free-ranged had significantly greater contributions of environmental microbes in their gut and skin microbiomes, suggesting that interactions with environmental communities can shape animal-associated microbiomes^7,34^. The wild ferrets in this study inhabited underground burrows and were likely exposed to rich, complex soil microbiomes. The reproductive microbiomes of wild ferrets were enriched for members of Actinobacteriota, an abundant phylum in soil microbiomes^50,51^. Specifically, wild members of both sexes showed enrichment for Actinobacteriota ASVs in genera such as *Blastococcus*, *Nocardioides*, and *Rubrobacter*, all of which are common soil bacteria, including in grassland soils^52^. In contrast, the ex-situ ferrets in this study were housed in environments that are cleaned and disinfected regularly, likely limiting or altering interactions between ferret and environmental microbiomes (Supplementary Fig. 1). Interestingly, however, ferrets at NZCBI were housed in enclosures with dirt/gravel floors. Yet there was no evidence of enrichment of soil microbes in their reproductive microbiomes, suggesting that it is not just mere exposure to soil but a more complex interaction unique to wild ferrets that shapes potential environmental contributions to reproductive microbiomes. Whether these environmental microbes are resident or functional members of the reproductive microbiomes is unclear, yet their consistent presence within wild ferret microbiomes suggests a possible interaction between host and environmental microbes, particularly in wild settings. Nevertheless, differences between the reproductive microbiomes of ex-situ and in-situ ferrets were not limited to soil-associated microbes, indicating that exposure to environmental microbiomes is not the sole driver of variation across ferret populations. Increased sampling of wild ferrets is needed to determine whether these patterns are robust across wild ferret populations.

Ex-situ members of both sexes harbored significant abundances of *Lactobacillus* (up to 35% assigned to this genus), and it was the only genus present from the family Lactobacillaceae. Although this does not nearly approach the *Lactobacillus* dominance found in human vaginal microbiomes, it is more abundant than previously reported in other non-human mammals. In domestic dogs, for instance, only 0.03% of the vaginal microbiome was identified as *Lactobacillus*^53^. Similarly low abundances of *Lactobacillus* have been reported in the vaginal microbiomes of non-human primates^7,54^, and domestic cattle and sheep^12,55^. In this study, however, the abundance of *Lactobacillus* was significantly lower in the microbiomes of wild ferrets (up to 5%) compared to ex-situ ferrets, suggesting an environmental influence. In humans, low vaginal pH, high glycogen in the vaginal epithelium, and high starch diets are all posited to facilitate *Lactobacillus* dominance^12^. Although vaginal pH of black-footed ferrets has not been reported, a previous study showed that the majority of animals had higher vaginal pH compared to humans^6^, suggesting that vaginal pH in black-footed ferrets is unlikely to facilitate *Lactobacillus* abundances. There is, however, variation in diet between ex-situ and in-situ ferret populations. Namely, ex-situ black-footed ferrets are fed combinations of commercial carnivore diet and whole rats or mice whereas wild black-footed ferrets rely heavily on live prairie dogs (*Cynomys* spp.) as their main food source. These different diets likely vary in nutrients and animal fibers, but their impact on reproductive microbiomes is unknown. Greater investigation of the physiological and environmental drivers of *Lactobacillus* abundance in non-human vaginal microbiomes may shed light on its role in promoting vaginal health and reproductive success. Moreover, previous work in black-footed ferrets has demonstrated that dietary vitamins A and E are casually linked to variation in sperm motility^56^ and, in humans, diet represents an established mechanism underlying reproductive microbiome variation^57,58^. Thus, diet provides an opportunity for future experimental work on prebiotic diets and their potential influence on microbiomes and reproduction in a conservation-focused setting^38^.

Components of black-footed ferret reproductive microbiomes were significantly associated with reproductive outcomes, particularly in ex-situ female ferrets. In regard to ex-situ females, and supporting our prediction, the two females that produced entire litters of non-viable kits had strikingly distinct vaginal microbiomes characterized by high phylogenetic diversity and disparate composition. As both individuals were sampled prior to pairing or breeding, it is possible that these signals of vaginal microbiome imbalance contributed to the future still-births. It is also possible that these females’ microbiomes were influenced by sexual activity (pairing and breeding) that occurred after our samples were collected, which we unfortunately cannot assess in this study. Unusually high vaginal microbiome diversity has been linked to pre-term and still births in humans^14,15,59^ and may be a biomarker of increased risk of negative reproductive outcomes for female ferrets. In one of these females (#9349), the vaginal microbiome was dominated by unidentified microbes and a member of the *Streptomyces* genus. *Streptomyces* is a known soil microbe but in rare cases, members of the genus can cause chronic bacterial subcutaneous infection (i.e., actinomycetoma) in humans and animals^60,61^. The prevalence of unidentified bacteria in the females with non-viable litters is difficult to interpret but it highlights the increased need to survey reproductive microbiomes in a wide range of animal hosts. Deeper sequencing (e.g., via shotgun metagenomics) of samples from a larger number of females with non-viable litters would enable greater characterization of these distinct communities and allow for assessment of any functional abnormalities in the microbial communities. Interestingly, the female that gave birth to a mixed litter of non-viable and viable offspring did not harbor a distinct microbiome, indicating that not all non-viable births are characterized by unusual vaginal microbiomes. In combination with the results discussed above on female ferret vaginal microbiomes being phylogenetically similar, the differences in community composition between females with viable and non-viable offspring suggest that maintaining key taxonomic members of female vaginal microbiomes may be important for promoting reproductive success. In humans, probiotic treatment has been used successfully to treat bacterial vaginosis and vaginal microbiome transplantation has shown success at reversing dysbiotic vaginal communities^62,63^. Although significantly greater study is needed before these approaches can be considered for endangered species such as black-footed ferrets, they provide support for extending the use of microbial therapies into conservation efforts.

Gross variation in sperm concentration was correlated with abundances of certain microbial taxa in prepuce microbiomes, but not with measures of bacterial diversity or composition. Namely, increased *Lactobacillus* ASVs were correlated with greater sperm concentration, which aligns with previous studies in humans that show a protective effect of *Lactobacillus* on sperm cells^64^ (but see^65^ for contrasting results). However, not all *Lactobacillus* ASVs showed this correlation, suggesting possible strain-specific interactions. Interestingly, *Lactobacillus* members were previously found to have increased abundance in the gut microbiomes of male and female ferrets during breeding season (vs. non-breeding season)^66^, suggesting that *Lactobacillus* may interact with reproductive processes throughout the body. Certain *Prevotella* ASVs were similarly correlated with increased sperm concentration, which contrasts with human studies showing that greater *Prevotella* correlated with decreased sperm quality and number^67^. Although our small sample size for wild males limits our ability to make broad conclusions about ex-situ vs in-situ sperm concentrations, previous research indicates that wild-born progeny of reintroduced black-footed ferrets have improved seminal traits compared to ex-situ ferrets^68^. In combination with our finding that prepuce bacteria correlate with sperm concentration, these results suggest potential interactions between environment, reproductive microbiomes, and male fertility that warrant further study. Importantly, however, semen microbiomes are hypothesized to be sourced from numerous male body sites (e.g., testicles, urethra, prepuce; Contreras et al. ^65^). Thus, prepuce microbiomes may be more strongly influenced by sexual activity and may not always mirror semen microbiomes in structure or function. Although semen samples are difficult to collect, particularly from endangered species and wild animals, studying male reproductive microbiomes in greater numbers across diverse populations, in combination with sperm quality metrics, can provide avenues for understanding and potentially modulating variation in male fertility.

In conclusion, our data on male and females black-footed ferrets provide evidence that their reproductive microbiomes are shaped by multiple factors (e.g., sex and environment) and that these communities may contribute to or at least reflect patterns of fertility. Variation between the sexes within a given location suggests potentially different physiological regulation of male and female reproductive microbiomes across ex-situ and in-situ settings, which mirrors sex-specific patterns reported in black-footed ferret gut microbiome^66^. Female vaginal microbiomes were found to be richer, but limited to phylogenetically similar microbes. Across environments, however, wild ferrets of both sexes harbored disparate microbiomes from their ex-situ conspecifics. These communities included microbes potentially derived from the environment, reinforcing previous studies^34,48,49,69,70^ and suggesting important, yet undefined, interactions between wild ferret and natural soil microbiomes. Nevertheless, sampling greater numbers of wild ferrets is needed to better elucidate variation across populations and environments, including in relation to environmental microbes. Finally, the results from males and females suggest preliminary correlations between reproductive microbiomes and fertility, including indicators of neonate survival. More research is needed to understand the potential of reproductive microbiomes as biomarkers of reproductive success in black-footed ferrets and other endangered species. Namely, future research should consider the interactions between male and female reproductive microbiomes (via e.g., copulation) as these dynamics have been shown to influence animal reproductive communities in both sexes^4^. These results provide the steppingstones for future targeted studies and approaches to microbial characterization and manipulation. For example, functional metagenomic approaches would provide further, valuable insight into the specific roles of potential biomarker taxa identified here (e.g., *Lactobacillus*, soil-associated bacteria, and unidentified taxa in females with non-viable offspring) and, in turn, suggest avenues for microbial manipulations that further conservation breeding efforts. In addition, previous conservation breeding programs have relied heavily on host genomic data to inform pairing decisions; we suggest that a multi-omics approach, including microbiome data, may further advance conservation breeding efforts in a more holistic manner. Ultimately, these results reinforce the urgent and growing call to better incorporate microbiome research into conservation efforts for black-footed ferrets and countless other endangered species reliant on ex-situ breeding for survival^71,72^.

## Methods

### Study subjects and sample collection

Reproductive microbiome swabs (male prepuce and female vaginal) were collected from 62 black-footed ferrets housed at NZCBI or FCC, or living in the wild at Conata Basin in Buffalo Gap National Grassland in Wall, South Dakota. In Conata Basin and the adjacent Badlands National Park, ferrets were reintroduced from 1994 to 1999 and established a free-ranging, self-sustaining population without any further releases from captivity. Wild ferrets are mainly fossorial, inhabiting underground tunnels and burrows in prairie dog “towns”. Ferrets at NZCBI were housed individually either in indoor or outdoor enclosures with dirt/gravel floors and ALPHA-dri bedding with nest boxes that are disinfected weekly. FCC ferrets are housed individually in indoor enclosures of metal and plastic substrates with ALPHA-dri bedding. Ferrets at both ex-situ facilities were fed similar commercial carnivore diets (e.g., Milikin meats Toronto Zoo blend) and whole prey (processed or live mice, rats, and hamsters). To avoid seasonal confounds, all samples were collected between March and May in 2021 and 2022. We have complied with all relevant ethical regulations for animal use and report our IACUC permits below.

Male prepuce swabs were collected by swabbing the opening to the prepuce (i.e., internal penile sheath) while rotating the swab for 5–10 s. Female samples were collected by swabbing the vulvovaginal region while rotating for 5–10 s. A single swab of the same type (551C Nylon-Flocked Dry Specimen Collection Swab Tubes, Copan Diagnostics, CA, USA) was collected for each animal. Samples were collected when animals were in hand and/or under anesthesia either for routine veterinary procedures at NZCBI and FCC (NBFFCC IACUC# 2022-1 and 2022-4) or for population surveys and health monitoring at Conata Basin (USFWS permit NBFFCC IACUC# 2022-3). Swabs from NZCBI were stored at −20 °C immediately upon collection and, within 2 h, were transferred to −80 °C for storage until DNA extraction. Swabs from FCC and Conata Basin were stored at −20 °C immediately upon collection and were stored there until being shipped on dry ice to the NZCBI’s Center for Conservation Genomics (Washington, DC) where they were stored at −80 °C until extraction.

Reproductive outcome data for ex-situ females included the total number of live offspring produced prior to sampling and number of non-viable offspring produced within 6 months before or after sampling. Among females for which these data were available, the total number of live offspring produced prior to sampling ranged from 0 to 15 (Supplementary Table 1). Across both facilities, ten females produced viable offspring while 14 did not produce any offspring. Three NZCBI females produced non-viable offspring within 6 months of microbiome sampling. Although there were estimates for the number of surviving offspring in the year following sampling for Conata females (as discussed in the Discussion), it was not possible to confirm as accurate number of viable or non-viable offspring for wild females so we did not analyze those estimates in this study. For ex-situ and wild males, we included sperm concentration (million cells per milliliter; *n* = 21 males), which was determined from semen samples collected during electroejaculations performed during veterinary procedures within 6 months of microbiome sampling (USFWS permits TE064682-1, TE-704930-2).

### DNA extraction, library preparation, and sequencing

Genomic DNA extractions were performed using the QIAcube HT platform with the DNeasy PowerSoil Pro QIAcube HT kit (Qiagen, Germany). Slight modifications to the manufacturers protocol to improve DNA yield included additional incubation steps prior to bead-beating (65 °C for 10 min at 40 rmp) and prior to the final elution (C6 warmed to 60 °C and added to samples to incubate for 5 min). We extracted multiple negative (unused swabs and empty tubes) and positive controls (ZymoBIOMICS microbial community; Zymo, Irvine, CA, USA; Catalog No. D6300) in parallel with the ferret samples. Extracted DNA was quantified using Qubit 1X dsDNA High Sensitivity Assay Kit (ThermoFisher Scientific, Waltham, MA, USA).

We prepared 16S rRNA gene metabarcoding libraries for the V3–V5 region (515F-Y and 939R primers) using a one-step PCR library preparation procedure^66,73^. PCR reactions included: 12.5 μL KAPA HiFi HotStart ReadyMix (Roche Molecular Systems, Inc., Indianapolis, IN, USA), 4.5 μL water, 1 μL bovine serum albumin (20 mg/mL), 1 μL barcoded forward primer (10 μM), 1 μL barcoded reverse primer (10 μM), and 5 μL DNA for a total reaction volume of 25 μL. Cycling conditions included: 1 cycle of 95 °C for 3 min; 25 cycles of 98 °C for 20 s, 62 °C for 15 s, 72 °C for 15 s; and 1 cycle of 72 °C for 1 min. Each sample was run through PCR in duplicate and the PCR products were visualized via agarose gel before the duplicates were combined to minimize PCR artifacts and maximize yield. We included negative (sterile double-distilled water) and positive (ZymoBIOMICS microbial community; Zymo, Irvine, CA, USA; Catalog No. D6305) PCR controls. We cleaned the PCR libraries (Apollo 324 System; IntegenX Inc., Pleasanton, CA, USA), quantified them using Qubit (1X dsDNA, high sensitivity, ThermoFisher Scientific) and qPCR (KAPA Library Quantification Kit for Illumina platforms, Roche Molecular Systems), pooled in equimolar ratios, and sequenced them on identical, duplicate Illumina MiSeq runs (2 × 300 bp paired-end) at the NZCBI’s Center for Conservation Genomics Laboratory.

### Data processing, bioinformatics, statistics, and reproducibility

A bioinformatic pipeline using a combination of commands in QIIME2 (v. 2023.1) and RStudio (R v. 4.1.0) was run on the demultiplexed data generated by the Illumina MiSeq platform. For data from each of the two runs, we used dada2 in QIIME2 with identical parameters to quality filter and trim sequences, merge forward and reverse reads, and remove chimeric sequences^74^. Data from the two runs were then merged by sample. We assigned taxonomy using a Naïve Bayes classifier pre-trained on SILVA v. 138.1 99% full-length sequences^75,76^ and generated ASV feature tables for the merged data. In addition to taxonomic identifications provided by SILVA, we applied the NCBI BLASTn tool to query sequences from ASVs of analytical interest against NCBI GenBank’s 16S ribosomal RNA sequences database.

Using the frequency and prevalence methods (based on our negative control samples) in the *decontam* package in R we removed 197 potential contaminant ASVs^77^. ASVs identified as non-bacterial (Archaea, chloroplasts and mitochondria) and those not assigned at Kingdom level to Bacteria were removed. We omitted ASVs with raw counts of less than ten reads across all samples. Samples with less than 1000 total reads (*n* = 3) were removed from downstream analyses due to insufficient sequence coverage. The ZymoBIOMICS microbial community standards (positive extraction and PCR controls) reflected the reported membership and composition as described by the manufacturer. The resulting, final feature table included 59 ferret samples with a total of 549,676 sequences (mean = 9317, range = 2943–83,723) representing 2478 ASVs.

To normalize sequence counts for alpha and beta diversity analyses, we scaled with ranked subsampling in R (SRS^78^) using a normalization sequencing depth of 2943 reads, retaining all samples and 98.4% of global species richness. We used the normalized counts to calculate three measures of bacterial diversity (alpha diversity: ASV richness, Shannon diversity, and Faith’s phylogenetic diversity) and two measures bacterial composition (beta diversity: UUF and WUF). To assess community membership, we first calculated the relative abundance of all taxa and included the conglomerate “Other” to represent the rare taxa that had relative abundances <1%. These were used for visualization purposes only. Then, to account for the compositional nature of microbiome data, we applied centered log-ratio (CLR) transformations to raw sequence counts, which reflect log-transformed ratios of the raw sequence counts of each taxon over the geometric mean of all other taxa in the community^79^ and used those in statistical tests as specified below.

To test for variation in bacterial diversity and CLR abundances, we applied LMs on the full dataset and on subsets of the data for each sex. For the full dataset, we included location, sex, and their interaction as fixed effect variables. For the female dataset, we included location, number of viable offspring, and number of non-viable offspring as fixed effect variables. For the male dataset, we included location and sperm concentration as fixed effects. Age was initially included in all full models but was found to be non-significant in all cases. We additionally used Akaike Information Criteria to compare full models with and without age as a main effect. In all cases, the model without age was found to be the most parsimonious model (Supplementary Table 2) and, therefore, age was not included in the reported results.

To assess main effects from the LMs, we report results from ANOVA Sum of Squares Type II and/or Type III (with Helmert contrasts), which are best suited to our unbalanced sample design. If there was an interaction term included in the model and it was found to be significant via Type II analysis, we reported only Type III (i.e., because Type II assumes there is no interaction between variables). If there was no interaction term or the interaction term was non-significant, we only reported Type II results as they are more powerful and informative for main variable effects^80^.

To identify taxa (phyla, genera, and ASVs) that were differentially abundant across variables of interest, we used ANCOMBC^37,81^, which calculates a W statistic reflecting the number of times the log-ratio of a specific taxon to every other taxon was significantly different between the variables of interest. It also determined whether the taxon is differentially abundant due to being a structural zero, which indicates that the taxon is absent or very rare in at least one of the variable levels. We report differentially abundant taxa according to most conservative cut-off threshold (0.9; Supplementary Fig. 2). To minimize the risks of false positives due to rare taxa, ANCOMBC analyses of ASVs were performed on tables for each dataset that were filtered to include ASVs with over 100 total reads.

To test for variation in bacterial community composition (beta diversity) according to location, sex, and reproductive outcome variables, we used PERMANOVAs with distance matrices (UUF and WUF) (R, adonis2 in {vegan}^82^). To account for our unbalanced sample sizes, our PERMANOVA models assessed the marginal effects of the terms such that they were not tested sequentially (i.e., via the “by = margin” term in adonis2). PERMANOVA model structure mirrored the models described above for diversity. We used Principal Coordinate Analyses to visualize clustering of beta diversity.

### Reporting summary

Further information on research design is available in the Nature Portfolio Reporting Summary linked to this article.

## Supplementary information

Supplemental Materials

Reporting Summary

## Data Availability

The primary data underlying these analyses is deposited as follows: Metadata (including sex, sample type, sampling location, collection date, number of viable and non-viable offspring, and sperm concentration) and analysis scripts (QIIME2 and R): Open Science Framework project https://osf.io/aru45/?view_only=d396cff260c44b87b80866ac90541235. Raw DNA sequence reads and accession numbers: NCBI Sequence Read Archive (SRA) under BioProject PRJNA1067562.
