# Author Correction: Markers of fertility in reproductive microbiomes of male and female endangered black-footed ferrets (Mustela nigripes)

**DOI:** 10.1038/s42003-024-06793-3

**Published:** 2024-09-10

**Authors:** Sally L. Bornbusch, Alexandra Bamford, Piper Thacher, Adrienne Crosier, Paul Marinari, Robyn Bortner, Della Garelle, Travis Livieri, Rachel Santymire, Pierre Comizzoli, Michael Maslanka, Jesús E. Maldonado, Klaus-Peter Koepfli, Carly R. Muletz-Wolz, Alexandra L. DeCandia

**Affiliations:** 1 Center for Conservation Genomics, Smithsonian’s National Zoo & Conservation Biology Institute, Washington, DC USA; 2 Department of Nutrition Science, Smithsonian’s National Zoo & Conservation Biology Institute, Washington, DC USA; 3 Department of Biology, Georgetown University, Washington, DC USA; 4 Smithsonian-Mason School of Conservation, George Mason University, Front Royal, VA USA; 5 Center for Animal Care Services, Smithsonian’s National Zoo & Conservation Biology Institute, Front Royal, VA USA; 6 National Black-Footed Ferret Conservation Center, US Fish and Wildlife Service, Carr, CO USA; 7 Prairie Wildlife Research, Stevens Point, WI USA; 8 Biology Department, Georgia State University, Atlanta, GA USA; 9 Center for Species Survival, Smithsonian’s National Zoo & Conservation Biology Institute, Front Royal, VA USA

**Keywords:** Microbial ecology, Conservation biology

Correction to: *Communications Biology* 10.1038/s42003-024-05908-0, published online 23 February 2024

In this article the funding from the Smithsonian Women’s Committee was inadvertently omitted. The original article has been corrected.

