# Supplementary material for: Markers of fertility in reproductive microbiomes of male and female endangered black-footed ferrets (Mustela nigripes)
Source: Commun Biol. 2024 Feb 23;7:224. doi: 10.1038/s42003-024-05908-0 (PMC10891159; doi:10.1038/s42003-024-05908-0)

| FerretID | sex | Location | Sample date | Sampling depth (# of sequence reads) | Age (years) | # live offspring | # nonviable offspring | Sperm concentration (million cells/milliliter) |
| --- | --- | --- | --- | --- | --- | --- | --- | --- |
| 20_045 | F | Conata | 3/14/22 | 13800 | ~1.79 |  |  |  |
| 20_060 | F | Conata | 3/14/22 | 5929 | ~1.79 |  |  |  |
| 20_137 | F | Conata | 3/16/22 | 4451 | ~1.79 |  |  |  |
| 21_012 | F | Conata | 3/15/22 | 8385 | ~0.79 |  |  |  |
| 21_019 | F | Conata | 3/15/22 | 85003 | ~0.79 |  |  |  |
| 21_065 | F | Conata | 3/16/22 | 8184 | ~0.79 |  |  |  |
| 21_071 | F | Conata | 3/16/22 | 5226 | ~0.79 |  |  |  |
| 20_089 | M | Conata | 3/14/22 | 5753 | ~1.79 |  |  | 547.3 |
| 21_017 | M | Conata | 3/15/22 | 6670 | ~0.79 |  |  | 524.4 |
| 21_042 | M | Conata | 3/16/22 | 6244 | ~0.79 |  |  |  |
| 9855 | F | FCC | 5/26/22 | 17544 | 1.02 | 0 | 0 |  |
| 9874 | F | FCC | 5/26/22 | 8967 | 1.02 | 0 | 0 |  |
| 9898 | F | FCC | 5/26/22 | 5389 | 1.00 | 0 | 0 |  |
| 9951 | F | FCC | 5/26/22 | 10465 | 0.98 | 0 | 0 |  |
| 10001 | F | FCC | 4/14/22 | 21182 | 0.86 | 0 | 0 |  |
| 10012 | F | FCC | 4/14/22 | 15258 | 0.85 | 0 | 0 |  |
| 10047 | F | FCC | 5/26/22 | 7753 | 0.96 | 0 | 0 |  |
| 10080 | F | FCC | 5/26/22 | 10442 | 0.96 | 0 | 0 |  |
| 10154 | F | FCC | 5/26/22 | 8230 | 0.92 | 0 | 0 |  |
| 10169 | F | FCC | 5/26/22 | 10175 | 0.87 | 0 | 0 |  |
| 9813 | F | FCC | 4/14/22 | 10735 | 1.77 | 1 | 0 |  |
| 9493 | F | FCC | 5/26/22 | 5709 | 2.96 | 11 | 0 |  |
| 9193 | F | FCC | 4/14/22 | 13077 | 3.88 | 0 | 0 |  |
| 8935 | M | FCC | 4/13/22 | 8373 | 4.85 |  |  | 30.3 |
| 8965 | M | FCC | 4/14/22 | 15996 | 4.85 |  |  | 509.9 |
| 8996 | M | FCC | 4/14/22 | 8584 | 4.84 |  |  | 191.5 |
| 9066 | M | FCC | 4/11/22 | 7217 | 4.81 |  |  | 226.3 |
| 9190 | M | FCC | 4/14/22 | 5137 | 3.88 |  |  | 423.6 |
| 9214 | M | FCC | 4/11/22 | 11179 | 3.86 |  |  | 403.3 |
| 9241 | M | FCC | 4/14/22 | 8498 | 3.85 |  |  | 418.9 |
| 9261 | M | FCC | 4/13/22 | 11993 | 3.84 |  |  | 492.5 |
| 9324 | M | FCC | 4/13/22 | 13544 | 3.77 |  |  | 89.3 |
| 9371 | M | FCC | 4/11/22 | 6569 | 2.93 |  |  | 655.9 |
| 9388 | M | FCC | 4/11/22 | 5640 | 2.88 |  |  | 99.4 |
| 9443 | M | FCC | 4/12/22 | 21609 | 2.86 |  |  | 494 |
| 9522 | M | FCC | 4/12/22 | 11654 | 2.83 |  |  | 767.5 |
| 9614 | M | FCC | 4/13/22 | 9752 | 1.93 |  |  | 40.6 |
| 9705 | M | FCC | 4/11/22 | 18307 | 1.85 |  |  | 382.8 |
| 9883 | M | FCC | 4/12/22 | 8488 | 4.84 |  |  | 106.4 |
| 9976 | M | FCC | 4/13/22 | 9907 | 0.86 |  |  | 667.6 |
| 9492 | F | SCBI | 3/30/21 | 10118 | 1.80 | 1 | 4 |  |
| 9263 | F | SCBI | 3/30/21 | 13535 | 2.81 | 5 | 0 |  |
| 9314 | F | SCBI | 3/30/21 | 11752 | 2.76 | 5 | 0 |  |
| 9653 | F | SCBI | 3/30/21 | 16903 | 0.86 | 6 | 0 |  |
| 9482 | F | SCBI | 3/30/21 | 8570 | 1.81 | 7 | 0 |  |
| 9212 | F | SCBI | 3/30/21 | 8226 | 2.82 | 8 | 0 |  |
| 9681 | F | SCBI | 3/8/22 | 14767 | 1.77 | 9 | 0 |  |
| 9332 | F | SCBI | 3/30/21 | 6114 | 2.70 | 10 | 0 |  |
| 9396 | F | SCBI | 3/30/21 | 12218 | 1.84 | 15 | 0 |  |
| 9729 | F | SCBI | 3/30/21 | 12792 | 0.81 | 0 | 2 |  |
| 9349 | F | SCBI | 3/8/22 | 8768 | 2.87 | 0 | 4 |  |
| 8974 | M | SCBI | 5/12/21 | 16948 | 3.92 |  |  |  |
| 9143 | M | SCBI | 5/12/21 | 10557 | 2.99 |  |  | 26 |
| 9186 | M | SCBI | 3/22/21 | 9906 | 2.82 |  |  |  |
| 9186 | M | SCBI | 5/12/21 | 40267 | 2.96 |  |  |  |
| 9341 | M | SCBI | 3/22/21 | 10533 | 1.99 |  |  | 3 |
| 9408 | M | SCBI | 3/30/21 | 12861 |  |  |  |  |
| 9431 | M | SCBI | 3/24/21 | 14278 | 1.81 |  |  |  |
| 9631 | M | SCBI | 3/30/21 | 33974 | 0.88 |  |  |  |

**Supplementary Table 1**. Metadata on black-footed ferrets (*Mustela nigripes*) from two *ex-situ* facilities (FCC and SNZCBI) and the wild (Conata). Ages for Conata ferrets are based on the assumption that they were born in late May/early June prior to sampling.

Supplementary Table 2. Akaike information criterion (AIC) values for comparisons of models with and without age as a fixed effect for alpha diversity linear models (ASV richness, Shannon diversity, and Faith’s phylogenetic diversity) and beta diversity PERMANOVAs (unweighted and weighted UniFrac diatnces). When model quality did not differ (AIC ∆<2), the most parsimonious model (i.e., without age) was reported.

| Linear model: ASV richness | AIC value |
| --- | --- |
| sex*location + age | 669.160 |
| **sex*location** | **667.212** |
| Linear model: Shannon diversity |  |
| sex*location + age | 183.874 |
| **sex*location** | **181.878** |
| Linear model: Faith’s phylogenetic diversity |  |
| sex*location + age | 565.103 |
| **sex*location** | **567.082** |
| PERMANOVA: unweighted UniFrac |  |
| sex*location + age | -94.888 |
| **sex*location** | **-95.647** |
| PERMANOVA: weighted UniFrac |  |
| sex*location + age | 22.841 |
| **sex*location** | **23.749** |

Supplementary Figure 1. Enclosures and environmental conditions experienced by black-footed ferrets (*Mustela nigripes*) at two *ex-situ* facilities (FCC and SNZCBI) and in the wild (Conata). Photos taken by co-authors and/or approved for use in this publication.


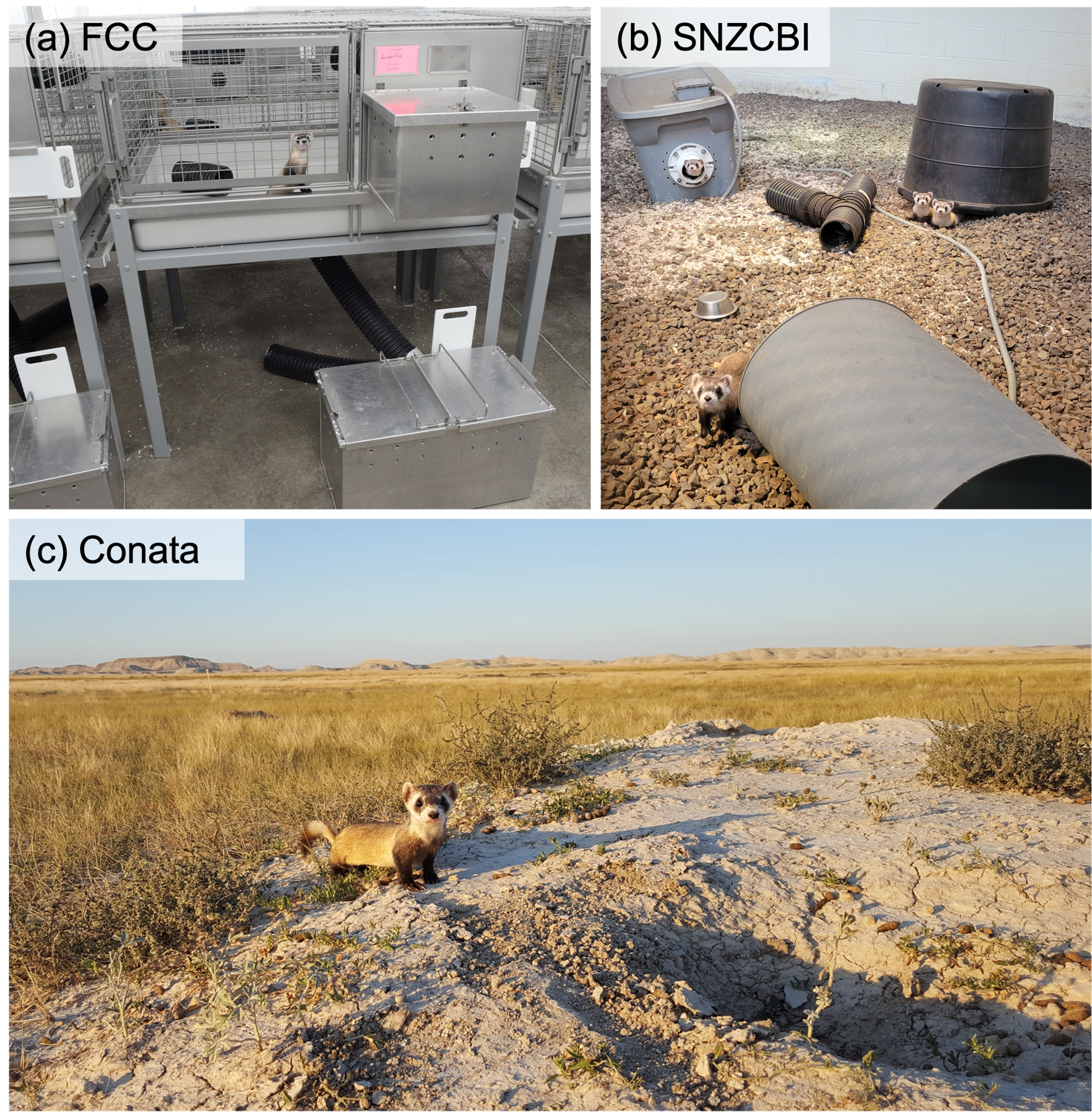


Supplementary Figure 2. Results of differential abundance testing using analysis of compositions of microbiomes with bias correction (ANCOMBC) between sexes at the (a) genus and (b) ASV level, as well as between locations in (c) males and (d) females.


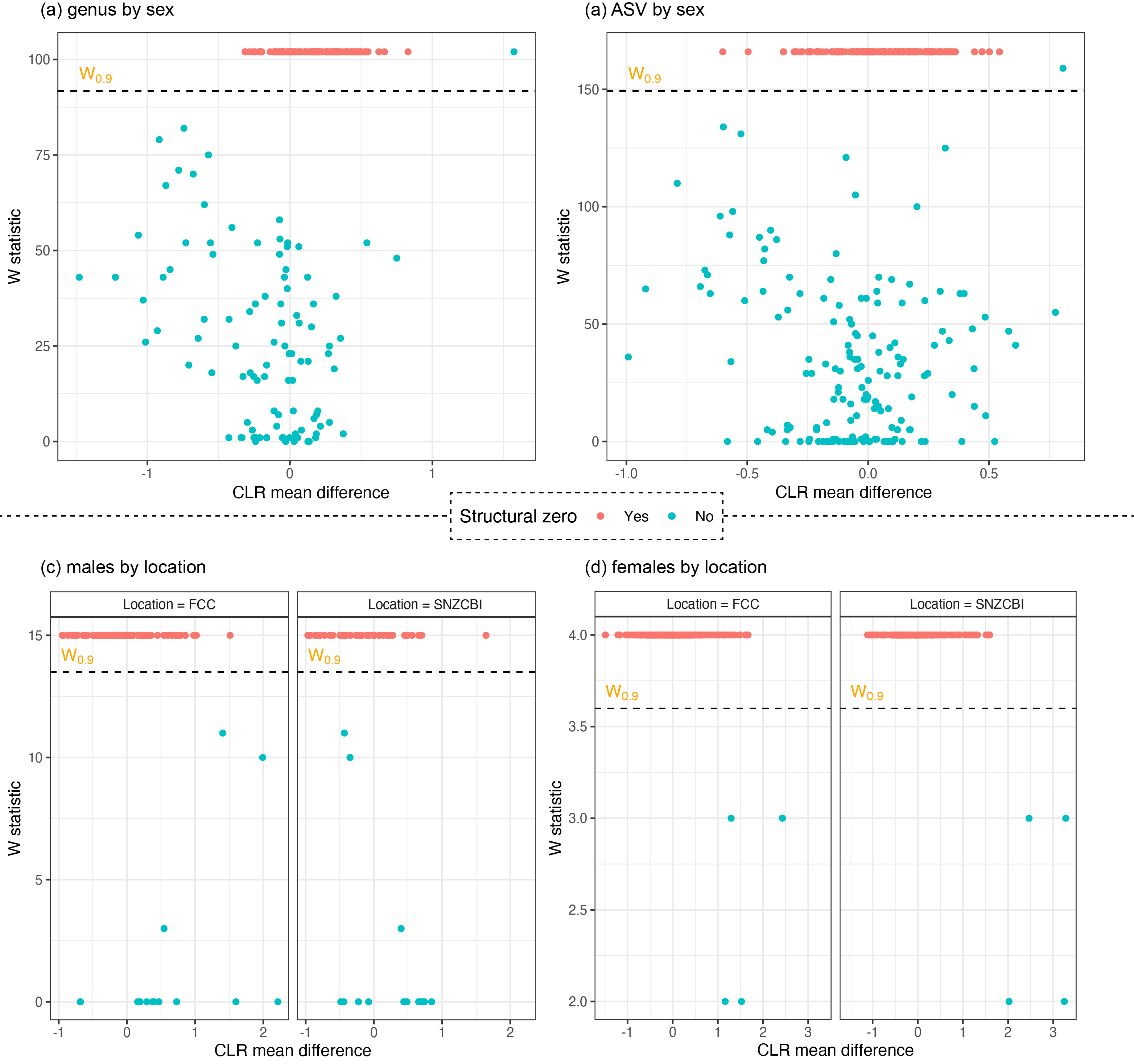

Supplement: Supplementary file 1 — Supplemental Materials [file 42003_2024_5908_MOESM1_ESM.docx]
