# Supplementary material for: Markers of fertility in reproductive microbiomes of male and female endangered black-footed ferrets (Mustela nigripes)
Source: Commun Biol. 2024 Feb 23;7:224. doi: 10.1038/s42003-024-05908-0 (PMC10891159; doi:10.1038/s42003-024-05908-0)
Supplement: Supplementary file 2 — Reporting Summary [file 42003_2024_5908_MOESM2_ESM.pdf]

nature portfolio

Corresponding author(s): DAPR COMMSBIO-23-3624-T

Last updated by author(s): Oct 17, 2023

Reporting Summary

Nature Portfolio wishes to improve the reproducibility of the work that we publish. This form provides structure for consistency and transparency in reporting. For further information on Nature Portfolio policies, see our Editorial Policies and the Editorial Policy Checklist.

Statistics

For all statistical analyses, confirm that the following items are present in the figure legend, table legend, main text, or Methods section.

n/a	Confirmed	
☐	☒	The exact sample size (*n*) for each experimental group/condition, given as a discrete number and unit of measurement
☐	☒	A statement on whether measurements were taken from distinct samples or whether the same sample was measured repeatedly
☐	☒	The statistical test(s) used AND whether they are one- or two-sided<br>*Only common tests should be described solely by name; describe more complex techniques in the Methods section.*
☐	☒	A description of all covariates tested
☐	☒	A description of any assumptions or corrections, such as tests of normality and adjustment for multiple comparisons
☐	☒	A full description of the statistical parameters including central tendency (e.g. means) or other basic estimates (e.g. regression coefficient) AND variation (e.g. standard deviation) or associated estimates of uncertainty (e.g. confidence intervals)
☐	☒	For null hypothesis testing, the test statistic (e.g. F, t, r) with confidence intervals, effect sizes, degrees of freedom and P value noted<br>*Give P values as exact values whenever suitable.*
☒	☐	For Bayesian analysis, information on the choice of priors and Markov chain Monte Carlo settings
☒	☐	For hierarchical and complex designs, identification of the appropriate level for tests and full reporting of outcomes
☐	☒	Estimates of effect sizes (e.g. Cohen's *d*, Pearson's *r*), indicating how they were calculated

Our web collection on statistics for biologists contains articles on many of the points above.

Software and code

Policy information about availability of computer code

Data collection	*Provide a description of all commercial, open source and custom code used to collect the data in this study, specifying the version used OR state that no software was used.*
Data analysis	Bioinformatics and statistical pipelines in QIIME2 (v. 2023.1) and RStudio (R v. 4.1.0).

For manuscripts utilizing custom algorithms or software that are central to the research but not yet described in published literature, software must be made available to editors and reviewers. We strongly encourage code deposition in a community repository (e.g. GitHub). See the Nature Portfolio guidelines for submitting code & software for further information.

Data

Policy information about availability of data

All manuscripts must include a data availability statement. This statement should provide the following information, where applicable:

- Accession codes, unique identifiers, or web links for publicly available datasets
- A description of any restrictions on data availability
- For clinical datasets or third party data, please ensure that the statement adheres to our policy

Upon acceptance, we will deposit the primary data underlying these analyses as follows:
• Metadata (including sex, sample type, sampling location, collection date, number of viable and nonviable offspring, and sperm concentration): Dryad and

Supplement
• Raw DNA sequence reads and accession numbers: NCBI Sequence Read Archive (SRA)

Research involving human participants, their data, or biological material

Policy information about studies with human participants or human data. See also policy information about sex, gender (identity/presentation), and sexual orientation and race, ethnicity and racism.

Reporting on sex and gender	*Use the terms sex (biological attribute) and gender (shaped by social and cultural circumstances) carefully in order to avoid confusing both terms. Indicate if findings apply to only one sex or gender; describe whether sex and gender were considered in study design; whether sex and/or gender was determined based on self-reporting or assigned and methods used. Provide in the source data disaggregated sex and gender data, where this information has been collected, and if consent has been obtained for sharing of individual-level data; provide overall numbers in this Reporting Summary. Please state if this information has not been collected. Report sex- and gender-based analyses where performed, justify reasons for lack of sex- and gender-based analysis.*
Reporting on race, ethnicity, or other socially relevant groupings	*Please specify the socially constructed or socially relevant categorization variable(s) used in your manuscript and explain why they were used. Please note that such variables should not be used as proxies for other socially constructed/relevant variables (for example, race or ethnicity should not be used as a proxy for socioeconomic status). Provide clear definitions of the relevant terms used, how they were provided (by the participants/respondents, the researchers, or third parties), and the method(s) used to classify people into the different categories (e.g. self-report, census or administrative data, social media data, etc.) Please provide details about how you controlled for confounding variables in your analyses.*
Population characteristics	*Describe the covariate-relevant population characteristics of the human research participants (e.g. age, genotypic information, past and current diagnosis and treatment categories). If you filled out the behavioural & social sciences study design questions and have nothing to add here, write "See above."*
Recruitment	*Describe how participants were recruited. Outline any potential self-selection bias or other biases that may be present and how these are likely to impact results.*
Ethics oversight	*Identify the organization(s) that approved the study protocol.*

Note that full information on the approval of the study protocol must also be provided in the manuscript.

Field-specific reporting

Please select the one below that is the best fit for your research. If you are not sure, read the appropriate sections before making your selection.

☒ Life sciences ☐ Behavioural & social sciences ☐ Ecological, evolutionary & environmental sciences

For a reference copy of the document with all sections, see nature.com/documents/nr-reporting-summary-flat.pdf

Life sciences study design

All studies must disclose on these points even when the disclosure is negative.

Sample size	59 black-footed ferrets
Data exclusions	Samples with less than 1,000 sequencing reads were removed from downstream analyses,
Replication	N/A. This study was not an experimental manipulation and therefore cannot be repeated. In addition, out study subjects are endangered animals and cannot be heavily manipulated or reproduced.
Randomization	N/A. This is a comparative study across animals of different sexes living in different environments. Our study subjects were determined by the availability of animals within those environments.
Blinding	N/A. Blinding was not necessary as there were no experimental groups.

Reporting for specific materials, systems and methods

We require information from authors about some types of materials, experimental systems and methods used in many studies. Here, indicate whether each material, system or method listed is relevant to your study. If you are not sure if a list item applies to your research, read the appropriate section before selecting a response.

Materials & experimental systems

n/a	Involved in the study
☒	☐ Antibodies
☒	☐ Eukaryotic cell lines
☒	☐ Palaeontology and archaeology
☐	☒ Animals and other organisms
☒	☐ Clinical data
☒	☐ Dual use research of concern
☒	☐ Plants

Methods

n/a	Involved in the study
☒	☐ ChIP-seq
☒	☐ Flow cytometry
☒	☐ MRI-based neuroimaging

Animals and other research organisms

Policy information about studies involving animals; ARRIVE guidelines recommended for reporting animal research, and Sex and Gender in Research

Laboratory animals	*For laboratory animals, report species, strain and age OR state that the study did not involve laboratory animals.*
Wild animals	Black-footed ferrets (endangered); Adults; housed at Smithsonian's National Zoo and Conservation Biology Institute in Front Royal, Virginia (SNZCBI; male = 17, female = 12) or the National Black-footed Ferret Conservation Center in Carr, Colorado (FCC; male = 23, female = 13), or living in the wild at Conata Basin in Buffalo Gap National Grassland in Wall, South Dakota (Conata; male = 6, female = 8). Samples were collected when animals were in hand and/or under anesthesia either for routine veterinary procedures at SNZCBI and FCC (NBFFCC IACUC# 2022-1 and 2022-4) or for population surveys and health monitoring at Conata Basin (USFWS permit NBFFCC IACUC# 2022-3). Following sample collection, all animals were either returned to their enclosures or released into the wild.
Reporting on sex	See above description for information on sex of animal individuals.
Field-collected samples	Male prepuce swabs were collected by swabbing the prepuce while rotating the swab for 5-10 seconds. Female samples were collected by swabbing the vulvovaginal region while rotating for 5-10 seconds. A single swab of the same type (551C Nylon-Flocked Dry Specimen Collection Swab Tubes, Copan Diagnostics, CA, USA) was collected for each animal. Swabs from SNZCBI were stored at -80C until DNA extraction. Swabs from FCC and Conata Basin were stored at -20C and shipped on dry ice to the SNZCBI's Center for Conservation Genomics (Washington, DC) where they were stored at -80C until extraction.
Ethics oversight	This research was approved by the USFWS Black-footed Ferret Recovery Program (NBFFCC IACUC# 2022-1 and 2022-4) and by the Smithsonian National Zoological Park Animal Care and Use Committee (NZP-IACUC Proposal #21-05).

Note that full information on the approval of the study protocol must also be provided in the manuscript.

Plants

Seed stocks	N/A
Novel plant genotypes	N/A
Authentication	N/A
